# A brief literature review and own clinical experience in prophylaxis of oral mucositis in children using low level laser therapy

**DOI:** 10.1051/bmdcn/2019090101

**Published:** 2019-02-22

**Authors:** Sergey Moskvin, Denis Pritiko, Elena Sergeenko, Elena Lukash, Leonid Gusev

**Affiliations:** 1 O.K. Skobelkin State Scientific Center of Laser Medicine under the Federal Medical Biological Agency Moscow 121165 Russia; 2 V.F. Voino-Yasenetsky Scientific and Practical Center of Specialized Medical Care for Children of the Department of Health of Moscow 119620 Russia

**Keywords:** Pediatric oncology, Oral mucositis, Low level laser therapy, Laser blood illumination

## Abstract

Objective: The article describes the experience of clinical application of low level laser therapy in pediatric oncology for the prevention and treatment of chemotherapy complications such as oral mucositis.

Background: For this purpose, for the first time in the world non-invasive laser blood illumination is used on the sinocarotid zone (on the projection of the common carotid artery symmetrically) and popliteal fossa in order to stimulate the phagocytic activity of leukocytes.

Methods: 25 children with different oncological diseases were given non-invasive laser blood illumination treatment (904 nm, pulse mode, 100 ns, 50-150 Hz, 5-7 W, 4 cm^2^, 1-2 minutes daily per each procedure) 1-3 days before the beginning of chemotherapy. For the objectification of the results of treatment, the phagocytic activity of leukocytes was evaluated.

Results: None of the children who underwent low level laser therapy course had any complications and no cases of oral mucositis developed.

Conclusions: Clinical experience has shown high efficacy and safety of low level laser therapy aimed at preventing the development of complications of chemotherapy, primarily oral mucositis, and stimulating the phagocytic activity of leukocytes.

## 1. Introduction

Oral mucositis (OM) - erosive and ulcerative lesions of the mucous membrane in the mouth, pharynx, esophagus, and in the whole gastrointestinal tract in adults and children, as a result of chemotherapy, radiation therapy of the head and neck area, as well as stem cell transplantation [1, 2]. Prevention and treatment of OM - including nutrition and pain management of patients with the severe form of mucositis - is part of the management in all children’s oncological clinics around the world, however, standard protocols for the treatment of this disease have not yet been developed. The frequency of relapses in patients with oncological diseases, even in the presence of preventive measures, can reach up to 85-100% after high-dosage chemotherapy and radiotherapy to head and neck tumors [3–7].

The possibility of using low level laser therapy (LLLT) for the treatment or prevention of the development of mucositis has been discussed by specialists for a long time, including the issue of a lack of evidence for the efficiency of laser illumination protocols [8, 9]. In a systematic study, J.M. Bjordal *et al.* (2011) [10] gives nearly exact recommendations of parameters of laser therapeutic techniques, but since there is little understanding of the mechanisms of the biological action of low-intensity laser light (LILL), the choice of wavelength and operating mode of the laser recommend by manufacturers of the apparatus unfortunately does not provide a satisfactory result. This raises quite justifiable doubts about the reproducibility of the data obtained [11]. The problem of misunderstanding the primary mechanisms of the biomodular action of LILL is particularly obvious, and can often be associated with “wandering in the dark” in search for the optimal parameters of low level laser therapy methods, which was manifested in the joint work of our Dutch colleagues [12, 13].

It is definitely time to learn how to choose the *optimal parameters for LLLT,* based on the requirements of biological and clinical feasibility, understanding the mechanisms of the biomodular action of LILL, rather than using whatever is first available from those who do not understand anything in the low level laser therapy methodology and for whom the laser device is only technical device, and the parameters, their rationale and purpose are absolutely incomprehensible and unknown.

We are also confident that the localization of the illumination zones (the basis of laser illumination techniques), used by all authors in relevant studies is an area of direct damage and is not optimal, since in the pathogenesis of mucositis, systemic disorders (not local pathological processes) prevail.

To date, a large number of reliable studies have been published, with respect to placebo, which prove the effectiveness of low level laser therapy for the treatment of children with mucositis after chemotherapy: a reduction in the likelihood of complications, the severity of the disease and the level of pain [14, 15]. For clarity, Table 1 presents the comparative data of several studies on the main indicator - complications alongside low level laser therapy and without it. We draw attention to the fact that most of the studies have been carried out for adults, not for children, but the overall picture is very obvious.

**Table 1:** The results of laser prophylaxis of oral mucositis after chemotherapy on patients with various oncological diseases.

The basic disease and methods of treatment	Age of patients, years	Laser therapy methods	Development of complications, %		Source
			After LLLT	Without LLLT	
A randomized, prospective, controlled trial during 93 sessions of chemotherapy with highdose methotrexate for acute lymphoblastic leukemia or lymphoma involved 33 children: 17 - main group, 16 - comparison group	The main, 1.7-17.9 (Av. age - 5.9) Comparison group 1.2-16.3 (Av. age - 8.4)	λ = 670 nm, HP, M = 30 mW, S = 0.5cm^2^; E = 12-24 secs per one zone. For prevention, each of the 13 affected areas were consistently illuminated: the left and right cheek tissues along the line of teeth closing, the retromolar space, the lateral and ventral surfaces of the tongue, and the palate, upper and lower lips.	59	88	Boris S.P. et al., 2016 [16]

A prospective, randomized, placebo-controlled study, 32 cycles of chemotherapy (21 for the treatment of osteosarcoma and 11 - acute high-risk lymphoid leukemia)	7-23 (Av. age - 14.6)	λ = 685 nm, M = 35 mW, HP, E = 54 secs per zone, 72 J/cm^2^. Punctually, perpendicular: jugal mucosa left and right (two zones on each side), the superior and inferior internal lip mucosa (one zone in each quadrant), the floor of the mouth (one zone on each side), the lateral edge of the tongue (two zones on each side), the tip of the tongue (one zone), the smooth palate (one zone on each side) and the labial commissure.	27	73	Abramoff M.M.F. et al., 2008 [17]

Randomized, double-blind prospective placebo-controlled Phase III study Chemo-radiotherapy for squamous cell carcinoma (nasopharynx, oropharynx and larynx) 94 patients: 47-group with LLLT, 47-group placebo.	LLLT therapy, 53.5 (±6.9) Placebo Group, 55.7 (±8.6)	For prophylaxis: λ = 660 nm, Power = 100 mW, Area = 0.24 cm^2^; E = 10 seconds per zone, 1J, 4 J/cm . In contact, nine zones per area: the mucosa of the lips, right and left buccal mucosa, the left and right lateral border of the tongue, buccal floor and ventral tongue. For treatment: in the placebo group, with symptoms of mucositis 3-4 grade): 660 nm, 100 mW, 1J, 8 J/cm^2^	40.4	78.7	Antunes H.S. et al., 2013 [18]

Randomized, double-blind, controlled trial chemotherapy (lung, GIT, skin, breast, lymphoma) 48 patients, 24 each in the LLLT and placebo groups	Group LLLT, 1772 (Av. age -44.5 ± 4.04) Placebo group, 18-79 (Av. age -46.2 ± 4.4)	λ = 630 nm, M = 30 mW, 5 J/cm^2^. 10 zones: two on the cheeks, two on the tongue, two on the floor of the mouth, one on the soft palate, one on the hard palate.	42	100	Arbabi-Kalati F. et al., 2013 [19]

Multicentre randomized phase III study of Radiotherapy (oropharyngeal, laryngopharynx and oral cavity carcinoma) 30 patients: 15 in the LLLT and placebo groups	36-78 (Av. age -60.4)	λ = 633 nm, M = 60 mW and 25 mW (1 patient), 2 J/cm^2^, E = 33 and 80 seconds (1 patient) per zone. 9 zones in the oropharyngeal area: posterior third of the inner surfaces of the cheeks, soft palate and anterior tonsillar pilllars.	7.6	35.2	Bensadoun R.J. et al., 1999 [20]

Randomized Study Chemotherapy or hematopoietic stem cell transplantation (HSCT). 60 children (leukemia, lymphoma, solid tumours): 29 - group LLLT, 31 - control group	3-18 (Av. age -8.7 ± 4.3)	λ = 780 nm, M = 60 mW, 4 J/cm^2^ On 5 areas: jugal mucosa, labial mucosa, edge of the tongue, soft palate, and sublingual region	22.5 (on day 8) (on day 15)	(on day 8) (on day 15)	Cruz L.B. et al., 2007 [21]

Chemotherapy (acute lymphoblastic leukemia) 40 children: groups of 10 people. preventive - A1 and A2, therapeutic B1 and B2	1-18	Prevention: A1: λ = 660 nm, M = 100 mW, S = 0.028 cm^2^, E = 10 secs per zone A2: λ = 830 nm, M = 100 mW, S = 0.028 cm^2^, E = 10 secs per zone Left and right jugal mucosa (two zones on each side), the superior and inferior internal lip mucosa (one zone in each quadrant), the floor of the mouth (one zone on each side), the lateral edge of the tongue (two zones on each side), the tip of the tongue (one zone), the soft palate (one on each side) and the labial commissure. Treatment: B1: λ = 660 nm, M = 100 mW, S = 0.028 cm^2^, E = 20 secs per point B2: λ = 830 nm, M = 100 mW, S = 0.028 cm^2^, E = 20 secs per point Directly, to the mucositis lesions	30 (A1) 50 (A2)	-	de Castro J.F.L. et al., 2013[22]

Double-blind, randomized placebo study Chemotheraoy (hemoblastosis) 55 patients: 27 - LLLT group, 28 - placebo	LLLT group, Av. age -27.3 ± 9.7 Placebo group, Av. age -29.7 ± 11	λ = 630 nm, M = 30 mW, continuous S = 1 cm^2^, 5 J/cm^2^ 10 zones in the posterior third of the inner surfaces of the cheeks, soft palate and anterior tonsillar pillars	31	41	Djavid G.E. et al., 2011 [23]

Triple-blind, placebo-controlled Phase III study Chemotherapy (head and neck cancer) Group LLLT-115, placebo-124	35-65 years old	λ = 633 nm, 24 mW/cm^2^, S = 1 cm^2^, E = 125 secs per zone, 3 J per zone 6 sites (borders of the tongue, floor of the mouth, buccal mucosa, labial mucosa, the soft palate and the oropharynx)	25	77	Gautam A.P. et al., 2013 [24]

Prevention. Prospective study Chemo (solid tumours) 26 patients	Av. age -51 (32-73)	λ = 650 nm, M = 100 mW and 780 nm, M = 50, 250 and 600 mW, S = cm^2^, E = 33 s per zone, 2 J/cm^2^	19	–	Genot- Klastersky M.T. et al., 2008 [25]
			
Treatment. A prospective, randomized trial Chemotherapy (hemoblastosis) 36 patients: group LLLT-18, placebo -18	LLLT group, Av. age - 56 (23-73), Placebo group Av. age - 44 (21-64)	Inferior and superior lips, right and left cheeks, right and left tongue, palate and velum palate, right and left gums, and tongue frenulum	17	89

Randomized double-blind phase III study Chemoradiotherapy (squamous cell carcinoma or undifferentiated carcinoma of the mouth, pharynx, larynx or metastasis in the neck with an unknown major cancer site) 75 patients: 37-group LLLT, 40- placebo	LLLT group, ± 9.4 (Av. age - 55) Placebo group, ± 10.3 (Av. age - 55.5)	λ = 660 nm, M = 10 mW, S = 4 mm^2^, E = 10 secs per zone, 2.5 J/cm^2^ 9 areas, punctually: the inferior and superior lips, right and left cheeks, dorsal and ventral part of tongue, hard and soft palate, right and left gums and tongue frenulum	11-11-22 (on weeks 2-4-6)	13-32-24 (on weeks 2-4-6)	Gouvêa de Lima A. et al., 2012 [26]

Randomized Study Chemotherapy and/or radiotherapy (hematologic or onco-hematological diseases, allogeneic HSCT) 22 patients: 12 - group LLLT, 10 - group 2 (mouthwash with a special solution: 0.15g of benzidamine, 1.13g of nistatin, 2g of neututocain and 10ml of distilled water)	LLLT group, Av. age - 32.7 2nd Group, Av. age - 27.5	λ = 660 nm and 780 nm every other day, M = 25 mW, E = 10 s, 6.3 J/cm^2^. Contactly on mucosa	66.7	80	Khouri V.Y. et al., 2009 [27]

A placebo-controlled, randomized trial Chemotherapy or HSCT 21 children: group LLLT-9, placebo-12.	Av. age - 8.2 (±3.1) LLLT group, 9.0 ± 3.3 Placebo group, 7.8 ± 3.0	λ = 830 nm, M = 100 mW, 4 J/cm^2^. The main zones: the floor of the mouth and the lateral/ventral part of the tongue	11.1	75	Kuhn A., 2009 [28]

Randomized controlled unilateral blind study HSCT 25 patients: group LLLT-11, control group -14	LLLT group, 36.8 ± 17.3 Control group, 36.6 ± 12.5	λ = 660 nm, M = 40 mW, S = 0.04 cm^2^, E = 4 secs per zone, 0.16 J, 4 J/cm^2^. 10 zones per area: mucosa of the lips, right and left buccal mucosa, lateral part of tongue to the right and left, ventral part of tongue and buccal floor	27.2	57.1	Silva G.B.L. et al., 2015 [29]

Av. age: average age; LLLT: low level laser therapy; λ: wavelength; CW: continuous wave; P: power; S: area; E: exposure (time)

Having considerable experience in providing specialized medical care to children with cancer, we have always focused our efforts on improving the methods of low level laser therapy. Statistics of the last six years showed that alongside preventive courses of low level laser therapy, OM developed in only 14% of children (without the courses - up to 80%), allowing us to reduce the inpatient stay of children on average by 4-5 days, and reduce the cost of treatment by up to 10 times [30]. In the course of our work, we also came to the conclusion that the percentage of complications can be reduced almost to zero, if we optimize the low level laser therapy methods (protocol of treatment). The search for ways to reduce the frequency of OM development during chemotherapy led us to study changes in the phagocytic activity of leukocytes as one of the common mechanisms of the biomodular action of LILL [31], under the influence of the non-invasive laser blood illumination technique (NLBI).

## 2. Case Reports

Between April 2017 and March 2018, 25 children received treatment: 6 children with osteogenic sarcoma aged 7 to 17 years (mean age 13.5 years), 4 children with Ewing sarcoma between the ages of 5 and 15 (mean age 9 years), 10 children with central nervous system tumors aged 1.5 to 10 years (mean age 5.2 years), 4 children with rhabdomyosarcoma aged 4 years (2) and 8 years (2), and one a child with a Wilms tumor that was six years of age. 18 patients had previously undergone high-dose polychemotherapy, which was complicated by the development of OM, and in 5 children chemotherapy was performed for the first time.

1-3 days before the beginning of chemotherapy, all children were assigned to be given NLBI treatment daily, using a known technique [32, 33], according to official clinical guidelines [34]. Children under 12 years - 1-2 procedures, older children - up to three.

**Fig. 1:**
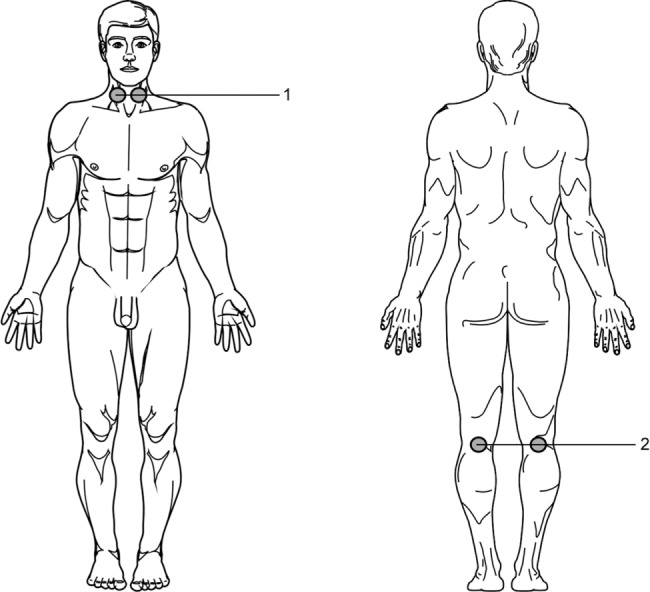
Impact localization.

Protocol of low level laser therapy (NLBI): wavelength 904 nm, pulse mode, light pulse duration 100 ns, frequency 50-150 Hz, pulse power 5-7 W, illumination area 4 cm^2^, exposure 1-2 minutes daily per each procedure. Illumination was carried out simultaneously by two laser emitting heads symmetrical to the sinocarotid zones (1, Fig. 1) or under the knee fossa (2, Fig. 1).

For the objectification of the results of treatment, the phagocytic activity of leukocytes was evaluated, because LILL, though a positive influence on this process, prevents the development of microflora, even with leukopenia [31, 35]. Phagocytic activity of leukocytes is also one of the factors of the state of nonspecific resistance of the organism.

Procedure for determining the phagocytic activity of leukocytes: Heparinized (50 *U/ml)* blood in a 0.05 *ml* amount was incubated in conical tubes at 37°C, with 0.05 *ml* washed with a latex suspension. After 5 minutes and after 1 hour, smears were prepared, which were colored according to Romanovsky-Giemsa. Then the phagocytic index (PI) - the percentage of phagocytic cells from 100 cells of phagocytes was calculated. PI is the number of cells from 100 counted cells involved in phagocytosis. For example: if only 20 cells out of 100 counted cells phagocytized latex particles, then FI is 20%. The phagocytic number (PN) - the average number of particles captured by one cell was also calculated.

## 3. Case report 1

Child P, 14 years old, entered the department of pediatric oncology with diagnosis: osteogenic sarcoma. During the preoperative preparation, the child received a high-dose of methotrexate - 12, 000 mg/m^2^, which was 15, 000 mg. Taking into account that at the first injection of the drug, oral mucositis of the 2nd degree developed in the child, and until the next dose the child was went through three procedures of the NLBI. From Table 2, it can be seen that LLLT allowed an increase of the percentage of active phagocytes, which persisted up to 10 days. Oral mucositis did not develop in the child.

**Table 2:** The study of the phagocytic activity of the leukocytes of the patient P., 14 years old.

Time of investigation of phagocytic activity	Time of incubation, min			
	5		60	
	PI, %	PN	PI, %	PN
Before low level laser therapy	24.00	11.7	44.00	16.6
Straight after 3 sessions of LLLT	68.00	7.5	73.00	13.9
10 days after LLLT	74.00	8.1	71.00	10.1

## 4. Case report 2

Child S, 7 years old, entered 9.10.17 in the department of pediatric oncology for carrying out programmed polychemotherapy (PCT) using the SIOPLGG2010 protocol. Anamnesis of the disease: sick since summer 2016, headaches, reduced visual acuity. MRI of the central nervous system dated 06.05.17 - multifocal tumor of chiasmal sellar area with spreading Cerebral Peduncle, amygdala and basal nuclei from both sides and optic nerves. The displacement of the median structures is 9 mm to the left. 05/23/17 - partial tumor removal (biopsy) - diagnosis: fibrillar astrocytoma (glioma of low grade). Clinical diagnosis: C71.8 Glioma of a low degree of malignancy of the chiasmatic-selar region, intracranial divisions of the optic nerves, subcortical structures, temporal region and islet region on the left. Condition after partial resection of the tumor. Continued tumor growth. The condition after performing the ventriculo-peritoneal bypass, the state at the time of the stage of the program cyclic PCT using SIOPLGG- 2010 protocol, class 2. The pronounced effect. Complications of the main diagnosis: G81.1 left-side hemiparesis. H47.2 organic damage of the conducting paths of the visual analyzer. H50.0 the strabismus is friendly convergent. H55 nystagmus is horizontal. K71.9 Hepatological toxicity of the 1st degree. Concomitant diagnosis: B18.2 viral hepatitis C.

12.10.17 - surgical operation was carried out. Implantation of the ventriculo-peritoneal shunt MiethkeproGAV (5) + ShuntAssi- sistant with the endoscopic installation of the ventricular catheter. Endoscopic septostomy. From 01.11.17 the child began to receive a cyclic PCT using the SI0PLGG-2010 protocol. The treatment was tolerated with grade 1 hepatological toxicity, with grade 2 vincristine neuropathy.

After 12 weeks of cyclic PCT as per protocol, a study was made of the phagocytic activity of leukocytes (Table 3), and taking into account the poor performance, four procedures of NLBI were prescribed.

**Table 3:** The study of the phagocytic activity of the leukocytes of the patient S., 7 years old. (the first study)

Time of investigation of phagocytic activity	Time of incubation, min			
	5		60	
	PI, %	PN	PI, %	PN
Before low level laser therapy	4	3.5	24	10.4
Second day after LLLT	16	11.8	44	15.3
5th Day after LLLT	18	10.6	54	14.1
10th day after LLLT	20	7.8	30	16.9

Table 3 shows that immediately after LLLT the phagocytic index increased almost 2-fold, and the phagocytic number increased 1.5-fold. The child underwent chemotherapy satisfactorily. Oral mucositis did not develop. An increase in phagocytic number was noted even after the end of chemotherapy.

Before the next course of PCT, the child was given only 2 procedures of the NLBI, since the indices of phagocytic activity were rather high. At a good level, these indicators remained after the termination of PCT (Table 4), which the child has transferred well. Oral mucositis did not develop.

**Table 4:** The study of the phagocytic activity of the leukocytes of the patient S., 7 years old. (the second study)

Time of investigation of phagocytic activity	Time of incubation, min			
	5		60	
	PI, %	PN	PI, %	PN
Before low level laser therapy	18	6	78	12.8
8th day after LLLT	12	8.8	74	16.9

Before the next course of PCT, there was only one LLLT session. In a smear made after five minutes of incubation of blood in a thermostat, phagocytosis was absent. After an hour of incubation, a very low phagocytic activity was noted in the smear. Immediately after the procedure, blood was taken for investigation and phagocytosis increased dramatically (Table 5).

**Table 5:** The study of the phagocytic activity of the leukocytes of the patient S., 7 years old. (the third study)

Time of investigation of phagocytic activity	Time of incubation, min			
	5		60	
	PI, %	PN	PI, %	PN
Before low level laser therapy	Phagocytosis is absent		20	3.4
One hour after LLLT	8	12.2	44	14.5
3 days after LLLT	64	12.2	100	13.7

The child underwent chemotherapy satisfactorily. Oral mucositis did not develop.

## 5. Case report 3

Girl M. 7 years old with a diagnosis of osteosarcoma of the right femur, T2N0M0, stage IIB, condition after combined treatment, clinical group II. According to vital indications, anticancer treatment was carried out. PCT using OS-2006 protocol was started on December 6, 17. The second course was held from 15.01 to 22.01.18. During the first two courses of PCT, even against the background of the ongoing medical decontamination, OM of the 1st degree developed. During the 3rd course of chemotherapy, including high doses of methotrexate, it was decided to conduct LLLT and analyze the dynamics of phagocytic activity after three 20-minute sessions of low level laser therapy. The 3rd course was conducted from 02.02.2018 to 23.02.2018 using 0S-2006 protocol: Methotrexate 12 g/m^2^ on the 1st, 8th days, IV infusor in 4 hours, single dose = 8 g, daily dose = 8 g; Cisplatin 50 mg/m^2^ on the 15th, 16th days, IV infusor in 24 hours, single dose = 35 mg, daily dose = 70 mg; Doxorubicin 45 mg/m^2^ on the 17th, 18th days, IV infusor in 24 hours, single dose = 31.5 mg, daily dose = 63 mg.

The girl underwent the treatment satisfactorily. Oral mucositis did not develop. It is noted that even 5 days after the termination of PCT, the child has high phagocytic activity. It increased more than 3 times compared with phagocytic activity before LLLT. High phagocytic activity was also noted 4 weeks after LLLT (Table 6).

**Table 6:** The study of the dynamics of phagocytic activity of the leukocytes of the patient M., 7 years old.

Time of investigation of phagocytic activity	Time of incubation, min			
	5		60	
	PI, %	PN	PI, %	PN
Before low level laser therapy	12	3.2	53	7
After 3 sessions of LLLT	20	5.4	72	8.3
8 days after LLLT	74	8.1	71	10.1
14 days after LLLT	48	8	64	12.4
18 days after LLLT	60	7.2	86	19
27 days after LLLT	72	5.8	82	15.5

We performed non-invasive laser blood illumination to children 1-3 days before the start of chemotherapy. Laser blood illumination was carried out by applying the emitter to the skin above large vessels. These can be zones of the carotid arteries and cubital, subclavian or popliteal veins.

## 6. Results

In total, in the group consisting of 25 patients, 47 procedures of NLBI were performed, and OM had developed in no cases, or other complications were recorded. Clinical experience has shown high efficacy and safety of laser light therapy aimed at preventing the development of complications of chemotherapy, primarily oral mucositis, and stimulating the phagocytic activity of leukocytes.

The number of procedures prescribed by the NLBI, first of all, depends on the initial index of phagocytic activity of leukocytes. At high values, 1-2 procedures are sufficient, and at low indices that number can be increased. Since LLLT is carried out once a day, the procedures are continued, respectively, for up to 3-4 days.

## 7. Discussion

As noted earlier, preventive courses of low level laser therapy do not allow oral mucositis to develop, thereby significantly reducing the cost of maintaining patients in the hospital and the total cost of treatment 30. The high efficiency of NLBI is explained, in our opinion, by several factors and is realized through numerous mechanisms of laser biomodulation.

The main reason for the development of toxic effects of chemoradiotherapy is the generality of the targets of cytostatic therapy, both in the tumor and in normal tissues. Developing with the use of cytostatic and radiation exposure, toxicity is actually a continuation of their therapeutic activity, which is realized through various mechanisms of cell damage: damage to the genetic apparatus of the cell; activation of processes of free radical oxidation; damage to cell membranes; violations of protein synthesis and cell division; disturbance of energy metabolism [36]. At the same time, numerous studies have shown that LILL prevents the apoptosis of cells [37, 38], enhances the proliferation of fibroblasts [39–41], keratinocytes [42, 43], endothelial cells [38, 44–46] and activates the antioxidant system [42, 47].

It is known that regulators of wound healing process are various growth factors such as tumor necrosis factor (TNF-a), basic fibroblast growth factor (bFGF), keratinocyte growth factor (KGF), etc., all of which, in turn, can be well controlled with the help of LILL [48–54].

Disinhibitory disorders with the suppression of nonspecific resistance of the organism are associated with Ca^2^+-dependent disorders of the thioldisulfide status, development of general and metabolic immunodepression [55, 56]. The results of the studies showed that the reaction of the organism to LILL is expressed in the phasic changes of the thiol-dependent components of the cells, the activity of various blood enzymes (Na+, K+ - and Mg-ATPase, lactate dehydrogenase, glucose-6-phosphate dehydrogenase, acetylcholinesterase, succinate dehydrogenase), and the reaction rate and strength its manifestations depend on the energy and, especially, the time parameters of the laser illumination technique [57].

In patients with oropharyngeal cancer receiving chemoradio- therapy, the balance of pro- and anti-inflammatory cytokines is disturbed in peripheral blood (by increasing IL-1, IL-6 and TNF-a content by 4-7.5 times) and immunoglobulins sIgA and IgA, which is a consequence of the development of the inflammatory process and compensatory reactions of the organism at the systemic and local level. At the same time, the concentration of immunoglobulin sIgA in salivary fluid decreases on average by 3040% in patients with mucositis of I-II degree and more than 2-fold in patients with oral mucositis of III-IV degree, which is one of the causes of dysbacteriosis (prevalence of candidiasis) in the oral cavity [36]. There is no doubt about the normalizing effect of LILL on the regulation of all the components of the immune system that provide anti-inflammatory effect of LLLT [58-65].

An important link in the pathogenesis of OM is the violation of the oral microbiocenosis: the number of colonies of nonhemolytic streptococcus, staphylococcus, enterobacteria in the mucosa increasing by an average of three times, and the largest growth (5 times) is noted in yeast fungi Candida albicans and Candida glabrata [36]. In this case, the bacteriostatic and bactericidal action of LILL on the microflora is well known [66]. Stimulation of phagocytic activity of leukocytes does not allow the development of a pathogenic microflora, thereby preventing the formation of colonies on the resulting ulcers of the mucous membranes of the oral cavity.

Numerous studies by Russian scientists have shown that the NLBI normalizes microcirculation, activates endotheliocytes, stimulating their functional activity by dilation and opening of the reserve capillaries, thereby providing oxygen access to the epithelial cells and promoting activation of cellular metabolism [31].

## 8. Conclusion

The experience of our work convincingly shows that LLLT with the correct setting of parameters of the technique allows one to almost completely exclude the development of OM in children after chemotherapy. The analysis of blood on the phagocytic activity of leukocytes in cancer patients makes it possible to determine the actual readiness of the organism for confronting infections. If it was previously impossible to predict the development of OM, with the introduction of a new, additional study, the effectiveness of the prognosis is now significantly increased, and a method of objective recommendation for the appointment of low level laser therapy procedures also appears.

If we talk about the very method of the NLBI, then it can be improved by also using pulsed (duration of the light pulse 100150 ns), not infrared (904 nm), but red (635 nm) LILL [31, 33].

Comparison of the effectiveness of prevention of OM by direct illumination of the LILL of the oral mucosa and NLBI clearly demonstrates the advantage of the latter method. From all that is demonstrated above, it follows that the prevention of oral mucositis should include the following measures:

1. Immediately upon admission to the hospital, the patient should be examined by a dentist and, if necessary, the oral cavity is sanitized.
2. Together with all other analyses in the department, it is also necessary to take blood for the determination of phagocytic activity of leukocytes. Based on the data received, a decision is made as whether to conduct NLBI and the number of necessary procedures.
3. With low phagocytic activity, the child is provided with prophylactic low level laser therapy sessions (NLBI), and with high-dose PCT it is possible to add medication decontamination of the oral mucosa.
